# Perception of pain and distress in intubated and mechanically ventilated newborn infants by parents and health professionals

**DOI:** 10.1186/1471-2431-14-44

**Published:** 2014-02-15

**Authors:** Luciana Sabatini Doto Tannous Elias, Amélia Miyashiro Nunes dos Santos, Ruth Guinsburg

**Affiliations:** 1 Universidade Federal de São Paulo and Professor at Faculdade de Medicina de Catanduva - Faculdades Integradas Padre Albino, Catanduva, SP, Brazil; 2 Division of Neonatal Medicine at Escola Paulista de Medicina, Universidade Federal de São Paulo, Rua Vicente Felix 77 apt 09, São Paulo, SP 01410-020, Brazil

**Keywords:** Pain, Distress, Stress, Newborn, Pain assessment

## Abstract

**Background:**

An understanding of perceptions of parents and health caregivers who assist critically ill neonates is necessary to comprehend their actions and demands. Therefore this study aim to analyze the agreement among parents, nurse technicians and pediatricians regarding the presence and intensity of pain and distress in mechanically ventilated and intubated newborn infants.

**Methods:**

Cross-sectional study comprising 52 infants and 52 trios of adults composed of one parent, one nurse technician, and one pediatrician who all observed the same infant. All infants were intubated and under mechanical ventilation and were not handled during the observations. Each newborn was simultaneously observed by the trio of adults for 1 minute to evaluate the presence of pain and distress. The intensity of pain and distress that the adults believed was felt by the infants was marked in a visual analogical scale. Adults’ agreement about the simultaneous presence of pain and distress in each infant was analyzed by marginal homogeneity and Cochran tests. The agreement about the intensity of pain and distress in each infant was studied by Bland-Altman plot and intraclass correlation coefficient (ICC).

**Results:**

The assessments of pain and distress were heterogeneous in all three investigated groups of adults as determined by the results of a Bland-Altman plot. The presence of distress was more frequently reported compared with pain (marginal heterogeneity, p < 0.01). The pain and distress scores in each adult group were not correlated as shown by ICC [parents, 0.36 (95% CI: 0.01-0.63); nurses 0.47 (0.23-0.66); pediatricians, 0.46 (0.22-0.65)].

**Conclusions:**

Adults systematically underscore pain in comparison to distress in mechanically ventilated newborns, without recognizing the association between them.

## Background

Intensive care has undergone several alterations over time as a function of increasing medical knowledge and technological advancement [1]. Caregivers must be sufficiently prepared and skilled to manage such advances but should not lose the focus of their efforts, namely, that the patient is the center and subject of care. In the case of severely ill newborns infants, care is provided by a healthcare team that must be able to understand the nonverbal messages sent by these patients. In this context, the perception and understanding of the reactions of neonates in pain requires much more than a mere glance [2].

The frequent exposure of ill neonates to pain, particularly with premature infants, occurs at a critical period during the structural and functional organization of the central nervous system. Painful stimuli and repeated and/or long-lasting stressors can result in functional alterations of neural circuits [3]. The permanent effects of a hostile environment and the repeated performance of painful and uncomfortable procedures on newborn infants may cause an imbalance of homeostatic mechanisms and consequently negatively influence their short-, mid-, and long-term outcomes [3-7].

One possible approach to the prevention of such consequences is to minimize patient distress and treat pain. Pain is an unpleasant sensory and emotional experience associated with actual or potential injuries or described in terms of such injuries. Pain is always a subjective issue. Each human being learns how to use this term on the grounds of his or her own experiences [8]. Distress in turn is the lack of comfort or relief, whereas comfort is defined as wellbeing and being at ease [9]. Adult caregivers of such infants should be able to distinguish between the presence of pain and distress and the circumstances under which pain triggers distress behaviors. Such clinical sensitivity is crucial when deciding on the need for measures to improve the comfort of critically ill infants or the use of pharmacological or non-pharmacological analgesia to provide suitable pain relief. According to Frank and Bruce [10], assessment of pain in non-verbal infants is based in the assumption that human beings are capable of reliably and objectively transforming the verbal or behavioral signals expressed in a variety of ways by another person into an objective representation of the signals. This objective representation will be transformed in a concept that may trigger a therapeutic approach.

In this context, whether the adults’ perception that a neonate is in pain or distress will trigger different actions regarding analgesia or comfort measures. Therefore, an understanding of the beliefs and behaviors of parents and health caregivers who assist neonates is necessary to comprehend their actions and demands in everyday clinical practice [11,12] in the neonatal intensive care setting and to design interventions to adjust such actions to the patients’ needs for comfort and analgesia. Thus, the present study sought to determine whether parents, pediatricians, and nurse technicians similarly assess the presence and intensity of pain and distress in intubated newborn infants on mechanical ventilation.

## Methods

This is a cross-sectional study with prospective data collection by a protocol complying with the national guidelines and rules for research with human subjects and was approved by the research ethics committees of the participating institutions. All adults signed an informed consent prior to participation in the research. Parents also signed an informed consent for their infants’ participation.

Inclusion and exclusion criteria for neonates, parents and health professionals enrolled in this study and the procedures related to the interviews about neonatal pain evaluation by the adults were previously described by our group [13]. In summary, newborn infants enrolled in this study met the following inclusion criteria: signed informed consent by parents; postnatal age of 24 to 96 hours old; placement in an incubator, and presence of gastric tube, peripheral and/or central venous access, and conventional mechanical ventilation by a tracheal tube, independent of the ventilator settings. Infants with congenital malformations or chromosomal syndromes were excluded from the study.

The interviewed adults were selected according to the following groups: **Group 1** – the mother or father of an infant who met the inclusion criteria provided they were not healthcare professionals and were present during the visiting hours (a convenience sample); **Group 2** – nurse technicians randomly selected among all those not assigned to provide care to the patient to be observed and who agreed to participate in the study; after assessing one of the patients in the study, the participating nurse technician was excluded from further assessments (nurse technicians are the main nursing workers in Brazilian Neonatal Intensive Care Units; they have at least 12 years of education and a Technical Professional Education Course in Nursing; nurse technicians are responsible for the main care of the patients under the supervision of a registered nurse with a university degree); **Group 3** – pediatricians randomly selected among all those not assigned to provide care to the patient to be observed; each doctor assessed only one infant.

The interviews with the trios of adults, each trio observing the same infant, were performed at one-hour maximum intervals, ensuring that the adults did not observe the same infant on different occasions. All adults answered a sociodemographic questionnaire and were then asked to face the infants and observe them for one minute. Patients did not receive any handling (painful or not) during this observation period. Time since last feeding and number of previous invasive procedures in the studied infants were not recorded since this study aimed to evaluate what was adults’ perception of pain and distress in the observed patients, without testing the real neonatal status.

The adults marked two vertical visual analog scales, one for pain and the other for distress, to indicate how much pain and distress they believed the infant felt. “Absence of pain” or “absence of distress” was written next to the bottom of a 10-cm non-numbered line, and “worst pain” or “worst distress” was written next to the top. The mark done by the observers was measured with a millimeter ruler and the number obtained was divided by ten. Therefore, the measure in centimeters of both visual analogue scales was considered as the pain and the distress scores. The absence of pain or distress was established when the adults marked the bottom of the visual analog scale (0 cm). The main researcher explained to all participants, in the same words, how to mark pain and distress in the respective visual analogue scales, but did not mention what they should consider as pain or distress, since their perception of these words was being tested. No training was made regarding the use of the scale prior to the interview.

Intraclass correlation test was used for inferential analysis to assess the homogeneity or heterogeneity of the assessments of pain and distress within each trio of interviewed adults per observed infant. The marginal homogeneity test was used to compare the qualitative assessments of pain and distress performed simultaneously by the same adult, and the differences were determined using Cochran’s test. To assess homogeneity in the quantitative assessments of pain and distress according to the visual analog scale results (measured in centimeters), linear correlations were applied using plots of the scores of pain versus distress attributed by the adults of each group separately (parents, nurses technicians, and pediatricians). Next, the heterogeneity in the quantitative assessments of pain and distress reported by each group of adults was evaluated using a classic Bland-Altman plot [14,15]. Finally, multivariate regression analysis was used to test the association of disagreement between the pain and distress scores with possible explanatory factors related to the characteristics of the infants and the adult observers.

To calculate the sample size, the need for 10 adult trios was considered for each characteristic of infants and adults to be assessed in order to perform the multivariate regression analysis [16]. Each trio was composed of one parent, one nurse technician, and one pediatrician. Because five characteristics of interest were initially included (adults: schooling and number of children; infants: gestational age, gender and type of delivery), a minimum sample of 50 infants assessed by 50 trios of adults (150 adults) was required.

The SPSS 16.0 software package was used for all statistical analyses, and the level established to reject the null hypothesis was 5%.

## Results

As previously described [10], a total of 54 newborn infants from the participating units met the inclusion criteria during the study period, and three adult observers (one parent, one nurse technician, and one pediatrician) were located in each case to assess the infant’s pain and distress concomitantly. Only two among such 54 infants were not assessed because the mother (one patient) or the doctor (one patient) refused to participate in the study.

Among the 52 studied infants, 35 (67%) were born by Cesarean section and 33 (64%) were male. The infants presented the following characteristics [expressed in mean (range)]: birth weight - 1530 g (605–4270), gestational age - 32 weeks (25–42), five-minute Apgar score - 8 (1–10), and postnatal age - 42 hours (24–96). The main diseases causing admission to intensive care were: lung problems in 34 (65%), early sepsis in 9 (17%), and hypoxic-ischemic encephalopathy in three (6%). All patients had a gastric tube and were under mechanical ventilation for an average of 33 hours (range: 9–94). Regarding analgesia and sedation, 47 (90%) infants were receiving continuous intravenous infusion of fentanyl and eight of these 47 neonates were also receiving continuous midazolan infusion. All neonates were not receiving enteral feedings during the study period.

The 52 newborn infants were assessed by 156 adults belonging to three groups: **Group 1** comprised two fathers and 48 mothers, **Group 2** consisted of 52 nurse technicians, and **Group 3** consisted of 52 pediatricians. In the two cases of pairs of twins (four infants), the mothers assessed each infant at different times. The overall characteristics of the interviewed adults are described in Table 1.

**Table 1:** Demographic characteristics of the three studied groups

	**Parents n = 50**	**Nurse technicians n = 52**	**Pediatricians n = 52**	**p**
Age (years)	28 ± 8	31 ± 8	31 ± 5	0.027
Females	48 (96%)	52 (100%)	44 (85%)	0.004
White race	23 (46%)	34 (65%)	45 (87%)	<0.001
Completed high school	10 (19%)	50 (96%)	52 (100%)	<0.001
Catholics	32 (64%)	21 (40%)	36 (69%)	0.007
Married	37 (74%)	24 (46%)	16 (31%)	<0.001
Number of children	2.2 ± 2.0	0.9 ± 1.0	0.2 ± 1.0	<0.001
U$ monthly *per capita*	313 ± 520	800 ± 370	2.600 ± 1.850	<0.001

Heterogeneity in the qualitative assessment of pain by each trio of adults was noted per infant evaluated. The intraclass correlation coefficient (ICC) for the 3 observations (parent, nurse technician and physician) of absence or presence of pain in the 52 evaluated infants was 0.066 (95% CI: -0.084 to 0.249; intergroup agreement if ICC >0.75) [10]. Regarding presence or absence of distress, ICC was 0.137 (95% CI: -0.029-0.322), indicating also disagreement in the groups of adult observers about their perceptions of neonatal distress.

With respect to the qualitative assessments of pain and distress performed by the three groups of observers (parents, nurse technicians, and doctors) for the 52 infants, Table 2 presents the results obtained for the evaluation of simultaneous presence of pain and distress, pain only, distress only, and absence of both pain and distress. According to the marginal homogeneity test, the proportion of positive assessments of distress was higher than the proportion of positive assessments of pain by parents, nurse technicians, and by doctors. All three groups of observers exhibited the same pattern of disagreement regarding the simultaneous assessment of the presence of pain and distress (Cochran test: p = 0.628): all adult groups marked higher scores for distress than for pain in the observed infants. Quantitative analysis of the linear correlation coefficient also provided evidence for a lack of correlation between the pain and distress scores attributed by each adult observer, with coefficients of 0.359 (95% CI: 0.007-0.632) for parents, 0.471 (95% CI: 0.227-0.659) for nurse technicians, and 0.461 (95% CI: 0.215-0.652) for doctors.

**Table 2:** Agreement among adults about the simultaneous presence of pain and distress in the observed newborn infants

	**Pain +**	**Pain +**	**Pain -**	**Pain -**	p-value*
	**Distress +**	**Distress -**	**Distress +**	**Distress -**	
**Parents**	12 (23%)	4 (8%)	23 (44%)	13 (25%)	0.001
**Nurse technicians**	16 (31%)	4 (8%)	24 (46%)	8 (15%)	0.001
**Pediatricians**	13 (25%)	6 (11%)	17 (33%)	16 (31%)	0.003
**Total**	41 (26%)	14 (9%)	64 (41%)	37 (24%)	

* Test of marginal homogeneity.

**Figure 1:**
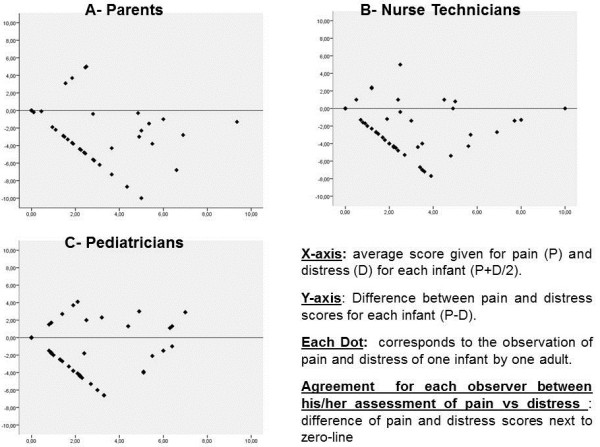
Heterogeneity in the evaluation of parents (Chart A), nurse technicians (Chart B) and pediatricians (Chart C) regarding the simultaneous presence of pain and distress in each one of the 52 newborns by Classic Bland-Altman plot.

To investigate the disagreement found among the adults regarding the presence of pain versus distress in the infants, a classic Bland-Altman plot was constructed (Figures 1A, B, and C). In these plots, the x-axis represents the average scores reported by each observer for pain and distress, and the y-axis, the difference between the scores of pain and distress (“pain score minus distress score”). When the assessment of pain and distress exhibited agreement, the scores for both variables were similar, and the difference between them tended to zero. Figure 1A reveals a lack of agreement in the assessments performed by parents regarding the simultaneous presence of pain and distress in each of the 52 infants. Negative values predominated because in 35 (67%) of the 52 assessments performed by parents, the magnitude attributed to distress was greater than the magnitude attributed to pain. The same pattern of disagreement was observed for nurse technicians (Figure 1B) and pediatricians (Figure 1C).

Finally, multivariate regression analysis was used to test the association between the characteristics of the observed infants and adult observers with the disagreement found in the assessments of pain and distress by the adults. The measure of heterogeneity in the assessment of pain and distress represented by the average difference between the pain and distress scores reported by the adult observers was chosen as the dependent variable. The characteristics of the infants and the adult observers were chosen as independent variables. Initially, the regression model included all possible influencing variables, and all those with p > 0.25 were sequentially removed. In total, eight models were constructed, but no independent variable exhibited any association with the disagreement found in the assessments of pain and distress by the adults.

## Discussion

Neonatal pain should be judged over time, according to infant’s response to interventions, environment and their general state of well-being, but there is a lack of tools to evaluate pain over time during neonatal intensive care stay. Health professionals adapt scales designed for acute pain evaluation to make decisions about management in critically ill infants. The experimental paradigm used in this study tried to mimic this clinical situation: if a parent or a health professional observes briefly a newborn infant during intensive care, will this person evaluate the situation as painful or as distressing? The difference between both conclusions may have, as a consequence, more or less willingness to perceive neonatal pain and treat it. The results obtained here demonstrate disagreement among health caregivers and parents as to the intensity of neonatal pain and distress attributed by these groups of adults to the observed infants and showed that there was no correlation between the assessments of pain and of distress made by the adults, regardless of their profession. In addition, for all three adult groups, distress seemed to be systematically more present than pain in infants placed in incubators with venous access, gastric tubes and under mechanical ventilation. No characteristic of either the infants or the adult observers exhibited association with the heterogeneity found in the assessment of the presence of pain versus the presence of distress by the observers.

Definitions of pain and distress [8,9] are grounded on subjective sensations that are also broadly and quite nonspecifically described, although the notion of distress generally encompasses the notion of pain; that is, pain may be the cause of distress. The lack of correlation between the intensities of pain and distress attributed by the adults to the investigated infants is remarkable because it is expected that when adults perceive that newborn infants are feeling in pain, they would also perceive that patient is distressed. Such a lack of correlation between pain and distress and the systematic attribution of higher scores to the latter compared with the former appear to indicate an unwillingness by the adults to acknowledge that the therapeutic support measures applied to severely ill neonates may also cause pain.

The intraclass correlation coefficient for the three observations (parent, nurse technician and physician) of absence or presence of pain and distress in the 52 evaluated infants showed disagreement among the groups of adults about their perceptions of pain and distress. The methods applied in this study difficult the analysis of which adult of the trio is more prone to overestimate or underestimate the neonatal pain and/or distress. However the heterogeneity of impressions among adults responsible for neonatal care may probably bring communication difficulties for health teams, imposing obstacles to implement adequate strategies to minimize pain and distress in critically ill newborn infants.

The disagreement found among parents, nurse technicians and pediatricians on the presence and the intensity of pain and distress in infants supports the findings of previous studies reporting differences in the assessment of pain by adult observers regarding adults, children and neonates as a function of particular personal, professional, and emotional traits of the observers [12,17,18]. The three groups of adults were different in their general characteristics: parents were slightly younger than health professionals and had significantly less years of education. Also parents had a higher frequency of a stable partnership and more kids. There were more white catholic males among pediatricians, who also had a higher income and social class. However, the logistic regression analysis could not identify any adult or neonatal characteristic associated to the heterogeneous assessment of pain and distress in the studied group. Despite this finding, there is some suggestion that empathy of observers for pain may motivate actions consistent with their affective state [19], and this may be true also for distress situations. Interesting venues or research in this issue are ways to assess adults’ emotional willing to differentiate between their neonatal patients’ pain and/or distress and to make active interventions to alleviate them.

Adults who play important roles in the prescription of measures to afford comfort and/or pain relief to critically ill infants disagree as to the magnitude of the patients’ possible feelings; this fact also indicates disagreement with respect to the patients’ therapeutic needs. Such heterogeneity in impressions may make communication difficult among the adults whom, in the last instance, are charged with providing comfort and pain relief to the neonates. The routine and frequent use of validated instruments to assess pain in the neonatal intensive care unit may improve communication among caregivers and among health professionals and parents [20].

There are some limitations in the study. First of all, the visual analogue scales were not validated to adults’ evaluation of neonatal distress. However, the research designed claimed for a similar tool to analyze adults’ perceptions of a subjective state in a preverbal infant, and the use of the visual analogue scale provided a unique opportunity to analyze pain and distress perceptions in quantitative and qualitative ways. Second, the nurse technicians may not be representative of the nursing professionals all over the world, limiting the generalization of their results; but their inclusion the study allowed the observation of health professionals active at bedside care and that have a different prospective of the infants compared to pediatricians. Finally, experience does influence how clinicians assess pain, but our questionnaire did not include this variable, that can be only indirectly assessed by the young age of all health professionals. Despite these problems, the present study is the first in the literature that addresses the difficulty exhibited by adults in the assessment of the terms “pain” and “distress” for newborn infants. In addition, this investigation differs from prior studies published on the assessment of pain in the neonatal period [21,22] because the focus was not the quantification of pain on occasions where infants are subjected to procedures known to be painful but rather the investigation of homogeneity or heterogeneity in the assessment and quantification of pain and distress by adults observing infants enduring a routine practice in intensive care, namely, mechanical ventilation.

## Conclusion

According to Franck and Bruce [10], after several years of concerns on the poor integration of pain assessment in neonatal care, perhaps it is time to reflect on whether the problem is more than translating research into practice. The results of the present study point out to a difficulty experienced by adults in noticing pain in infants and attributing perceived distress to pain. Strategies to overcome this difficulty should be investigated in the context of neonatal intensive care.

### Ethics

The IRB of the institution (Comitê de Ética em Pesquisa da Universidade Federal de São Paulo) approved the prospective collection of data related to this study in August 3, 2001 (CEP# 650/01). The same IRB approved the retrospective analysis of the data previously collected in August 8th, 2008 (CEP# 1035/08). All research was performed in accordance with the Declaration of Helsinki.

## Abbreviations

ICC: Intraclass correlation coefficient.

## Competing interests

Nothing to declare. None of the authors received any reimbursement, fee, funding or salary from any organization that may gain or lose financially with the publication of this manuscript. None of the authors have stocks or shares in any organization that may gain or lose financially with the publication of this manuscript. We do not have any patent approved or applied related to this manuscript. There are none non-financial competing interests.

## Authors’ contributions

LSDTE participated in the study design, collection of data, discussion of results, statistical analysis, and writing of the draft and the final version the manuscript. AMNS participated in the study design, discussion of results, and writing of the final version the manuscript, and RG participated in the study design, discussion of results, statistical analysis, and writing of the draft and the final version the manuscript. All authors read and approved the final manuscript.

## Pre-publication history

The pre-publication history for this paper can be accessed here:

http://www.biomedcentral.com/1471-2431/14/44/prepub
